# Idiosyncratic selection of active touch for shape perception

**DOI:** 10.1038/s41598-022-06807-2

**Published:** 2022-02-21

**Authors:** Neomi Mizrachi, Guy Nelinger, Ehud Ahissar, Amos Arieli

**Affiliations:** Brain Sciences Department, Weizmann Institute of Science, 7610001 Rehovot, Israel

**Keywords:** Neuroscience, Sensorimotor processing, Somatosensory system

## Abstract

Hand movements are essential for tactile perception of objects. However, the specific functions served by active touch strategies, and their dependence on physiological parameters, are unclear and understudied. Focusing on planar shape perception, we tracked at high resolution the hands of 11 participants during shape recognition task. Two dominant hand movement strategies were identified: contour following and scanning. Contour following movements were either tangential to the contour or oscillating perpendicular to it. Scanning movements crossed between distant parts of the shapes’ contour. Both strategies exhibited non-uniform coverage of the shapes’ contours. Idiosyncratic movement patterns were specific to the sensed object. In a second experiment, we have measured the participants’ spatial and temporal tactile thresholds. Significant portions of the variations in hand speed and in oscillation patterns could be explained by the idiosyncratic thresholds. Using data-driven simulations, we show how specific strategy choices may affect receptors activation. These results suggest that motion strategies of active touch adapt to both the sensed object and to the perceiver’s physiological parameters.

## Introduction

Perception usually co-occurs with sensors’ movements^1–10^. Moreover, in primates, it has been established that hand movements are an integral component of tactile perception of objects’ features^8,11–19^. Yet, the nature of these movements and their dependency on other perception-relevant factors are not sufficiently characterized. In a seminal series of studies, Lederman and Klatzky introduced the first comprehensive description of active ‘Exploratory procedures’ (EPs) of 3D objects—stereotyped movement patterns having invariant characteristics^8^. The choice of a specific EP was found to depend on the desired object-related information (e.g., the object’s hardness, texture, or shape). For example, ‘pressure’ (applying force to an object against a resisting force such as by bending the object) was found to be the primary EP for evaluating hardness, ‘lateral motion’ (sideways movements between the skin and the object surface) for evaluating texture and ‘contour following’ (*CF*; maintaining of hand contact with the object contour) for evaluating the shape of 3D objects^8^. *CF* variants were further analyzed in consequent studies^20–22^.

Initial studies of planar (2D) objects revealed that humans adapt their movement patterns to the spatial characteristics of the scanned surfaces. Speed is adjusted to the spatial frequency^14^ and direction to the spatial orientation of the surface’s texture^23,24^. In these studies, adaptations of hand speed and direction resulted in affecting temporal cues^14,24^ and in maintaining specific sensory cues in a given ‘working range’, likely optimal for sensation, consistent with principles of closed-loop control^11,25–27^. Similar maintenance of ‘controlled variables’ has been observed in other tactile tasks, both in humans and rodents^17,28–30^.

The application of EPs is probably optimized by practice. While children use a variety of EPs in a given task, only the most efficient EP is typically applied by adults^31^. When exploring gratings, participants optimize their scanning direction during the final stage of exploration in a way that improves their performance^24^. People can also change their scanning strategy after being directly taught to do so^14^.

Overall, studies on active touch have been providing convincing evidence that hand movements are an integral part of tactile perception and that, specifically, their general patterns are adapted to the desired object-related information^8,32^. Yet, the nature of the fine patterns of hand movements, which determine the acquisition of fine tactile information, and the drives for their specific adaptations, had not been studied so far. We have thus designed an experiment that allows accurate tracking of hand motion in relation to object details at high speed and high resolution. We tracked the hands of participants while they perceived planar (2D) shapes and correlated the observed scanning patterns with the characteristics of the participants’ spatial and temporal tactile sensitivities.

## Results

**Figure 1:**
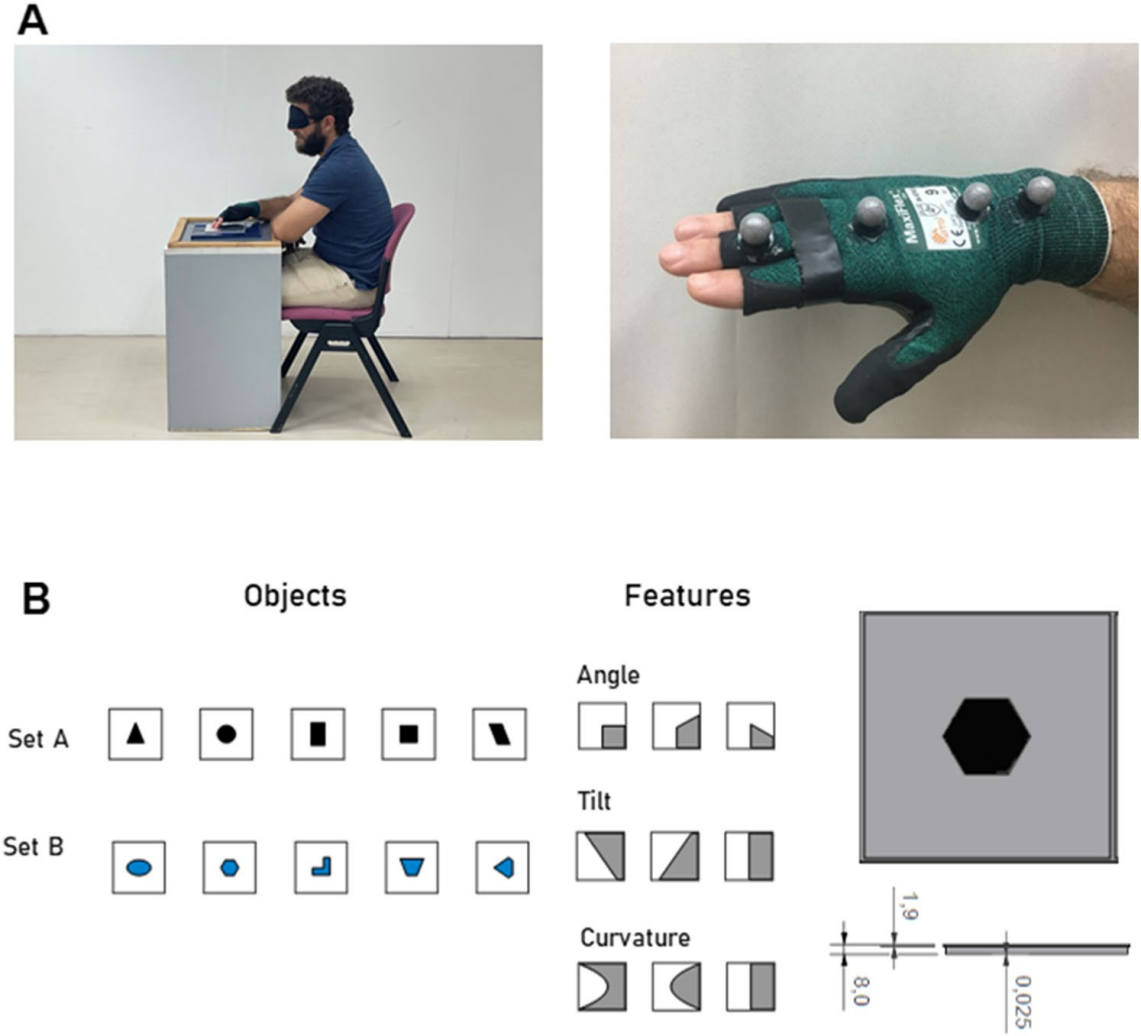
Recording and tactile items. (**A**) Left: participants were blindfolded and sat next to a table carrying the tactile object. Right: the glove used with four designated Vicon markers attached to it. Two markers were attached above the wrist carpal bones and two above the middle and proximal phalanx of the middle finger. (**B**) Tactile items. Right: two sets of tactile items were used: Objects (left) and Features (right). Objects were divided into set A (top, black) and set B (bottom, blue). Features were divided into three blocks: Angle (left), Tilt (middle) and Curvature (right). For all tactile items, the colored area was raised by 25 µm relative to the background area. Left: an illustration of the metal board that tactile items were engraved on (in this case—hexagon). Scale bar indicates height differences (mm).

Overall, 18,701s of hand movements were recorded at high resolution from 11 human participants while performing tactile recognition task of planar shapes (1196 trials in total). Separate finger movements were prevented by binding together the three palpating fingers (Fig. 1A). In order to generalize the results over the types of tactile items, two sets of tactile items were used—Objects (further divided to set A and set B) and Features (further divided to sets of Angle, Tilt and Curvature, Fig. 1B). In order to generalize over differences in idiosyncratic experience, three patterns of item-presentation orders were employed.

### Contour following and scanning are two common procedures for planar shape recognition

**Figure 2:**
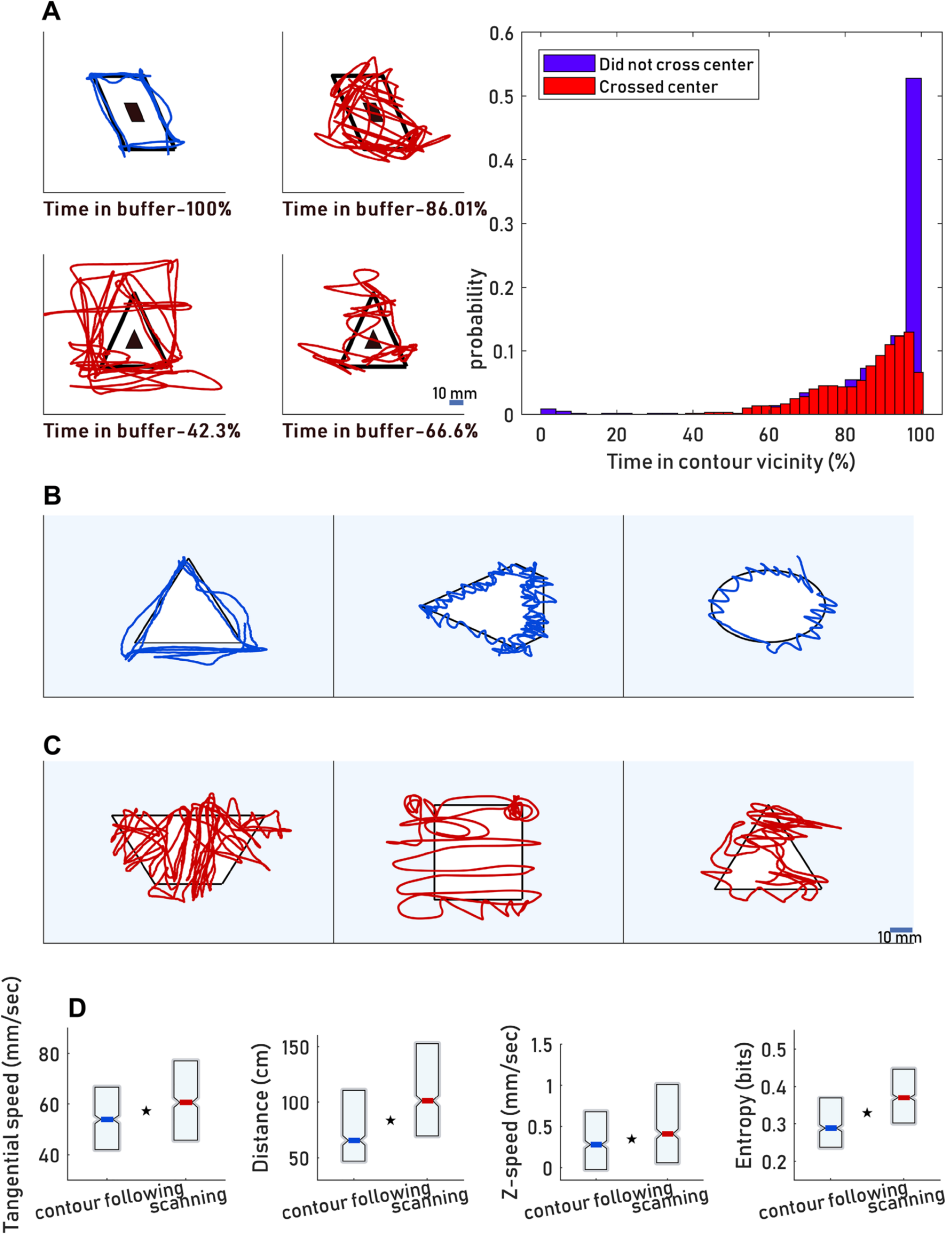
Classes of motor patterns. (**A**) Left: classification of individual trials was based on the degree of time spent in the shape center (left, small black shape) and in the contour vicinity (left, blue buffer between the inner and outer guiding shapes) (see “Materials and methods”). x–y plots of *CF* trials (left, blue) and *SC* trials (left, red) are depicted. Right: probability (y-axis) of the time spent in the contour vicinity (x-axis) based on whether the shape center was crossed or not. (**B**) *CF* trials were sub-classified as ‘*Linear*’ (left) ‘*Oscillating’* (middle) or both (right). (**C**) Examples of various manifestations of *SC* trials; common scale bar (C, right) for all panels. (**D**) Distributions (medians and quartiles) of four kinematic variables across all *CF* and *SC* trials (p < 0.001 for all differences). N_subjects_ = 11; N_Sessions_ = 51 (4–5 sessions per participant); N_trials_ = 1196. N_*CF* trials_: 537. N_*SC* trials_: 659.

In each trial, the trajectory of the hand was superimposed on the outline of the shape. The degree of time spent in the contour vicinity differed substantially across trials (Fig. 2A–C). Using a geometrical algorithm, we quantified the degree of coverage of the inner part of the shape area and the time spent in the contour vicinity (Fig. 2A right, black and blue areas, respectively; see “Materials and methods”). Approximately half of the trajectories did not cross between the shape contours while the remaining did (52% and 48%, respectively). In trials in which the trajectory did not cross the shape center, typically, most of the trial time was spent in the contour vicinity (Fig. 2A, right). Based on this analysis we have categorized the trajectories into two major classes: contour following (*CF*), where the trajectory mostly followed the shape’s outline and did not cross between them, and scanning (*SC*), where the trajectory either crossed between distant parts of the shapes’ contour or when a large portion of the trial time (≥ 25%) was spent far from the contour vicinity (Fig. 2A, left, red; see “Materials and methods”). *CF* was implemented in two main fashions: *Linear* motion (moving along the contour) and *Oscillating motion* (moving back and forth across the contour while advancing along it). Some *CF* trajectories included only *Linear* motion (Fig. 2B, left); others only *Oscillating* motion (Fig. 2B, middle); and others included both motion types (Fig. 2B, right). *SC* trajectories exhibited different degrees of shape coverage and different foci (Fig. 2C). On average *SC* trials differed from *CF* in their kinematics: *SC* trials were characterized by higher tangential (scanning) speeds, longer traveled distances, higher entropy and higher speeds along the z-axis (perpendicular to the scanned surface) (Fig. 2D, *p* < 0.001 for all differences, Mann–Whitney U test and Bootstrap). These strategy types and sub-types were exhibited for all objects and during all sessions tested in this study (Supplementary material, Fig. 1).

### Focal palpation

**Figure 3:**
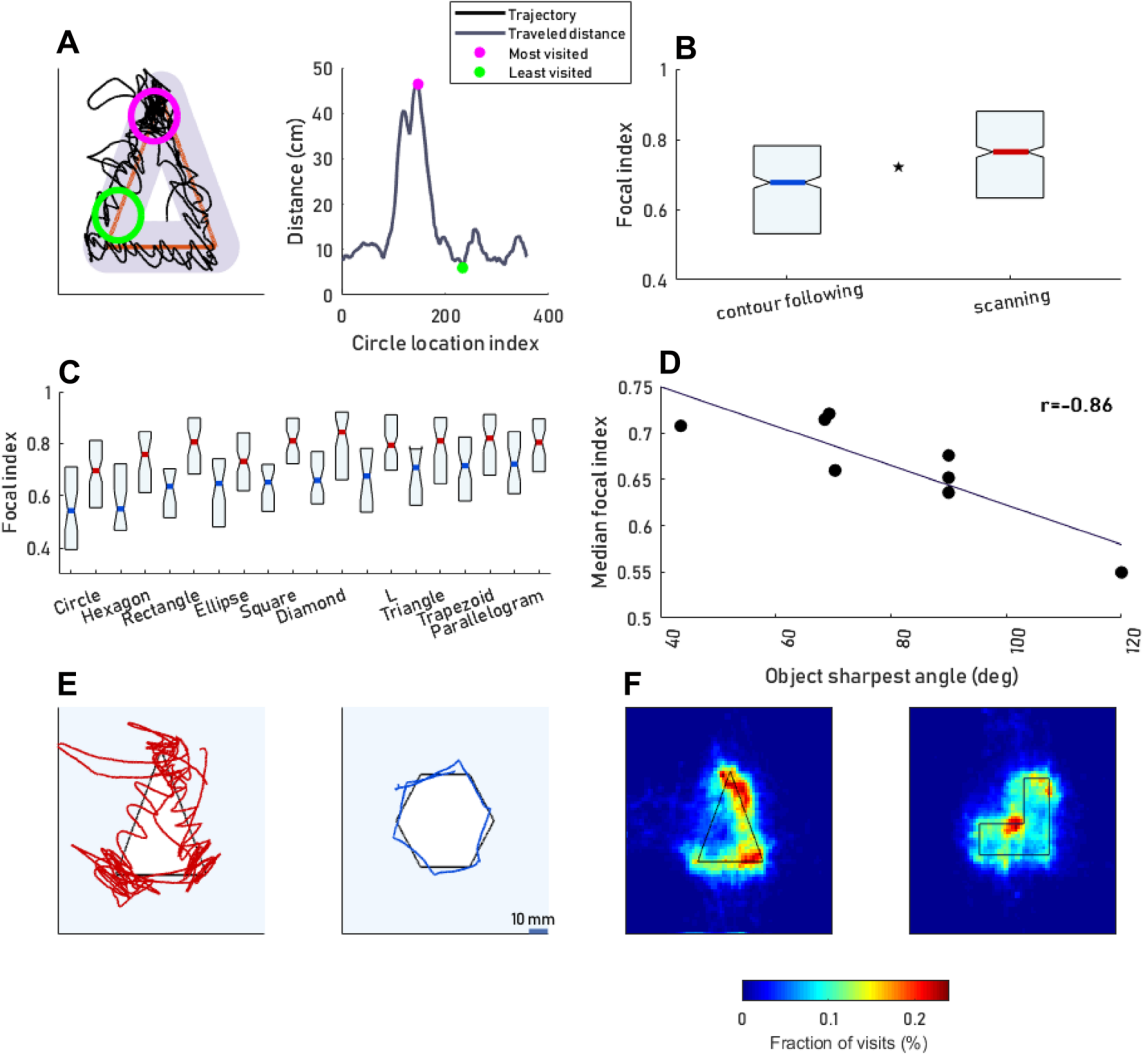
Focal palpation. (**A**) An example demonstrating the calculation of a trial’s focal index. Left: x–y plot of one example trial. Overlapping circles with a radius of 10 mm (gray) were plotted over the objects' outline (red). The traveled distance in each circle was calculated. Circles with the maximal and minimal value of traveled distance (pink and green, correspondingly) are marked. Right: calculated traveled distance (y-axis) as a function of the location of the circle’s center (x-axis). The trial’s focal index is defined as the ratio of the difference between the trial’s maximal and minimal values of traveled distance (pink and green) and their sum. (**B**) The distributions (medians and quartiles) of the focal index for *CF* and *SC* trials across all trials of all participants and all sessions (henceforth ’grand distributions’) (p < 0.001). Ns of subjects, sessions and trials are as specified in Fig. 2D. (**C**) Grand distributions of the focal index per object in *SC* and *CF* trials. (**D**) Each data point represents the object grand median focal index (y-axis) and the object sharpest angle (x-axis) for *CF* trials. Objects’ sharpest angle: Triangle = 43.5°, trapezoid = 68.2°, parallelogram = 69.2°, diamond = 70.3°, square, rectangle, L shape = 90°, hexagon = 120°.N_trials per shape_, panels B-D: Triangle, 70; Circle, 128; Rectangle, 68; Square, 83; Parallelogram, 80; Ellipse, 121; Hexagon, 66; L, 104; Trapezoid, 117; Diamond, 95. (**E**) x–y plots of two trajectories differing in their focal indices (Triangle, focal index (FI) = 0.77; Hexagon, FI = 0.34). (**F**) Mean visit rates for two example objects (triangle and L, all subject and trial types included).

A focal index (see “Materials and methods”) was used to evaluate the degree in which the participants exhibited non-uniform coverage of the shapes’ contours, focusing on specific parts of the explored shape (e.g., the part surrounded by a pink circle, Fig. 3A). *SC* trials’ median focal index was significantly higher than that of the *CF* trials (Fig. 3B, *p* < 0.001, Mann–Whitney U test and Bootstrap). This difference was evident for all objects of Sets A and B (Fig. 3C). A significant correlation was observed between the median focal index and the object’s sharpest angle (excluding the circle and ellipse) for *CF* trials (Fig. 3D, r = − 0.86, *p* = 0.006, *adjusted alpha* = 0.016; 0.05/3), but not for *SC* trials (*r* = − 0.69, *p* = 0.057, *r *_*Spearman*_ = − 0.65, *p *_*Spearman*_ = 0.08, *adjusted alpha* = 0.016; 0.05/3). No significant dependency was found (*p* = 0.33, Kruskal–Wallis test) between the focal index (per participant) and the order or type of items presented. Example trajectories of the objects with the highest or lowest sharpest angle (triangle and hexagon, respectively) are depicted in Fig. 3E. Visit rates (see “Materials and methods”) of two objects (Triangle and L shape), demonstrating the focus of specific shape regions, are depicted in Fig. 3F.

### Trajectories are idiosyncratic and depend on the explored shape

**Figure 4:**
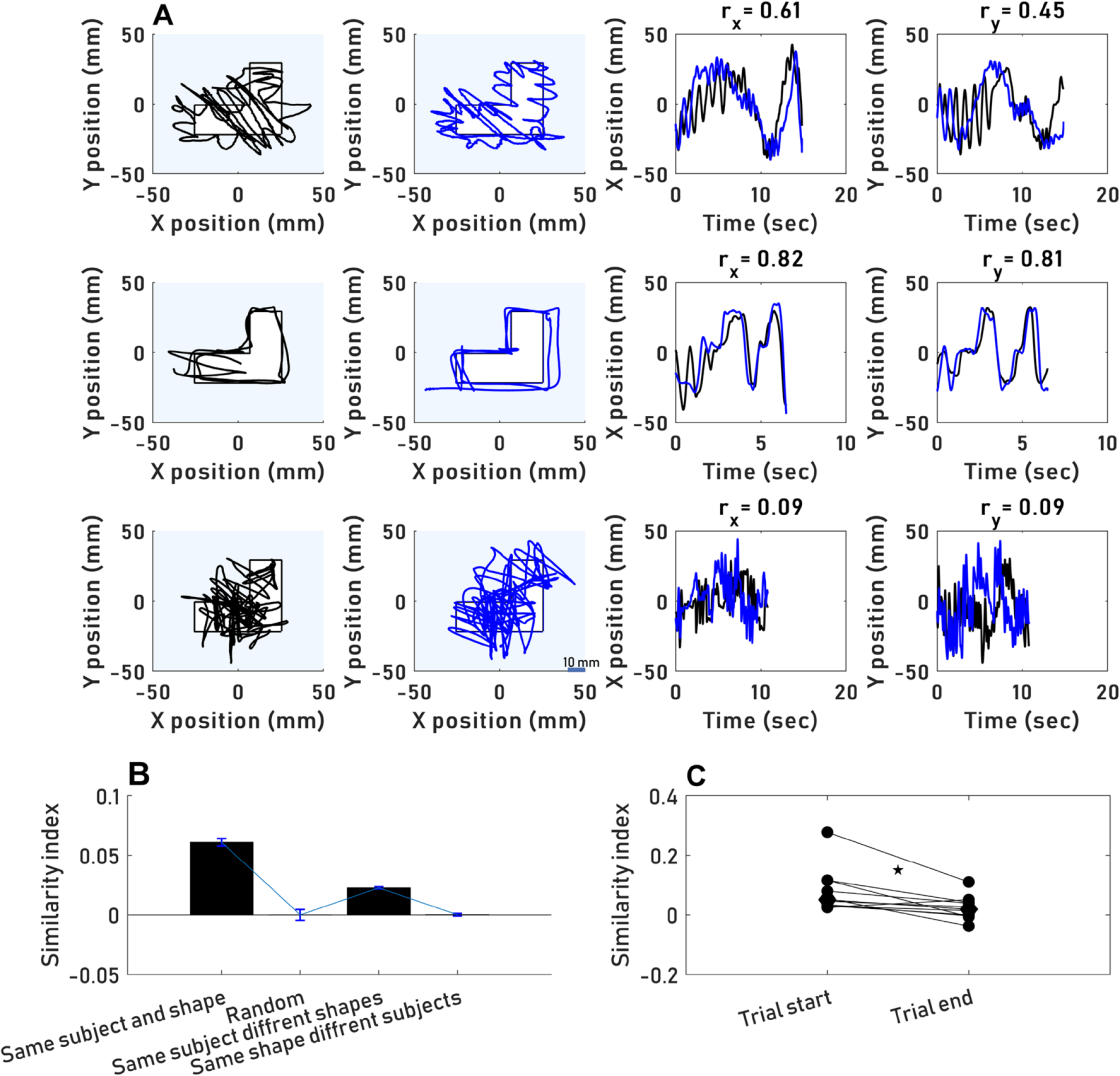
Idiosyncratic hand movements. (**A**) Three examples of three subjects’ hand trajectories while they explored the same shape (L). Top, subject EV; middle: subject AG, bottom, subject AS. Left: x–y plots for two sequential trials with the same object. Right: x and y positions projections (y-axis) as a function of time (x-axis), with the two trials superimposed after appropriate down sampling (see Materials and Methods). (**B**) Mean similarity indices (mean ± SEM across participants, see “Materials and methods”) for subject-shape pairs and control pairs. (**C**) Each circle represents the median similarity index of one participant in either the first half of the trial or the second. Median similarity indices across all participants are represented by the diamond shape. First-half median values were significantly higher (p = 0.005).

Previous studies described a repetition of eye movement’s trajectories in subsequent views of the same picture^33–37^. In order to test whether such repetition appears in hand movements we computed a similarity index for every pair of trials of a single participant when they explored the same shape (see “Materials and methods”). Similarity index values ranged between − 0.93 to + 0.96, exhibiting a bias towards positive correlations (*mean* ± *SEM*: 0.06 ± 0.003, *p* < 0.001, Mann–Whitney U test and Bootstrap). Two examples of strong and one example of typical positively correlated trajectories are depicted in Fig. 4A. These examples demonstrate that, as expected, the component that dominated the trial-to-trial similarity was the slow component of palpation; the rapid oscillations around the contours were typically not correlated across trials. The mean similarity indices of participants exploring the same shape were significantly higher than of controls which were close to zero or biased towards negative correlations; either of the indices of a random normal distribution, indices of the same participant exploring different shapes, or indices of different participants exploring the same shape (Fig. 4B, *p* < 0.001 for all differences, Mann–Whitney U test and Bootstrap). Similarity indices did not differ between *SC* and *CF* trials (*p* > 0.05, Mann–Whitney U test and Bootstrap). Similarity indices for the first half of each trial were significantly higher than those for the second half (Fig. 4C; *p* = 0.005, Wilcoxon signed-rank test). This difference is in line with previous reports, suggesting that initial eye movement patterns were replicated more often^38,39^. Between session I and III the similarity indices computed for the first half of the trials significantly increased (*p* = 0.048, Wilcoxon signed-rank test), suggesting that with practice, initial movements became more stereotyped. No significant dependency of participants’ similarity indices on the order or type of objects presented was found (*p* = 0.41, Kruskal–Wallis test).

### Palpation speed correlates with the participant’s idiosyncratic spatial resolution

**Figure 5:**
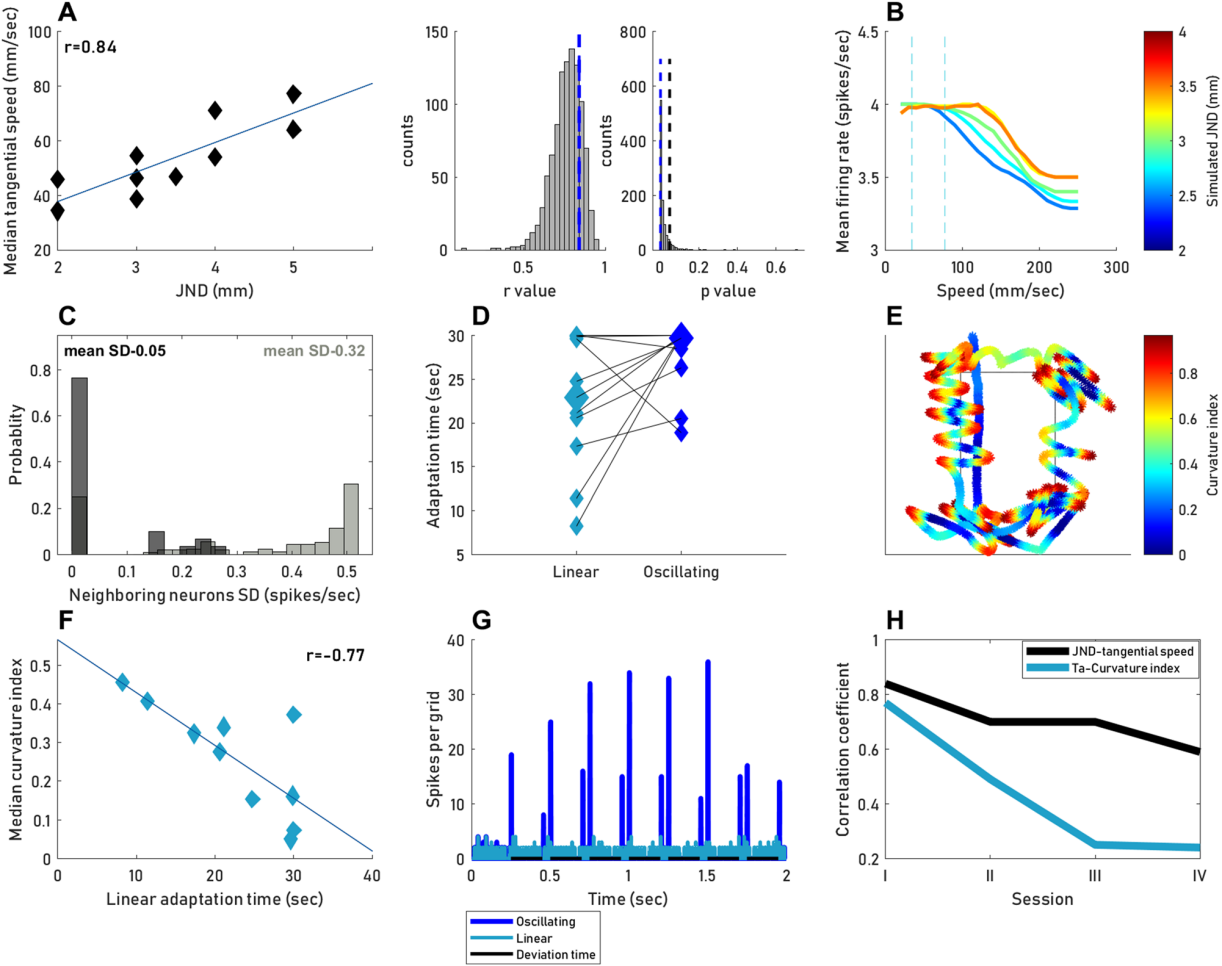
Dependency on sensory thresholds. (**A**) Left: each data point represents the median tangential speed (y-axis) across all trials of the first session and the middle finger JND (x-axis) of one participant (18–29 trials per participant). Right: probability distributions of r and p values across a bootstrap sample (N = 1000) of possible JND values, assuming known unreliability of JND measurements (see “Materials and methods”). Experimental r and p values are marked by the dashed blue lines. Black vertical dashed line represents *p* = 0.05. (**B**,**C**) TouchSim simulations (see “Materials and methods”). (**B**) The mean number of spikes per neuron (y-axis) plotted against tangential speed (x-axis). Color code represents the distance between units in the grid (2–4 mm). The range of speed used by our participants is marked by the dashed gray lines. (**C**) Histogram of SD values between firing rates of neighboring neurons, for either the window containing speed values used by our participants (dark gray) or all other equal-size windows (light gray). (**D**) Each two connected data points represent one participant’s mean adaptation time (Ta) using *Linear* (light blue) or *Oscillating* (blue) motion (11 *Linear* and 11 *Oscillating* trials per participant). Median values for each motion type are represented by a larger shape (median Ta  *Oscillating* = 29.07, median Ta *Linear* = 22.9 s). (**E**) Curvature approximation. The curvature index (see “Materials and methods”) during one example trial is color coded along the trajectory. (**F**) Each data point represents the median curvature (across all trials of the first session) and the mean *Linear* Ta of one participant (4–24 *CF* trials per participants). (**G**) Number of spikes (y-axis) over time (x-axis) for a grid of RA units for either *Oscillating* (blue) or *Linear* (light blue) motion. The time of deviation from the contour, in *Oscillating* motion, is marked in black. (**H**) Black: correlation coefficient between JND and tangential speed (y-axis) per session (x-axis). Light blue: absolute value of the negative correlation coefficient between the curvature and adaptation time (y-axis) per session (x-axis). N = 10 participants, for all panels.

With adaptive active sensing it is expected that scanning velocities are tuned to optimize receptor activation. It has been shown that primates prefer hand movement patterns that preserve certain temporal cues^13,14,23,24,40^, cues whose temporal frequencies best fit one class of mechanoreceptors in the fingertip^41–44^. Hence, adaptive active sensing predicts that variations in hand speeds across participants should correspond to variations in the spatial spacing of their receptors. In this case, the temporal frequencies of activation will be preserved in the preferred working range by the finger speed, as the temporal frequency generated on the skin when scanning a single edge is determined by the multiplication of the finger speed and the spatial frequency of receptors across the skin^45^. To test for this possibility, we measured the Just-Noticeable-Difference (JND) in the index, middle and ring fingers of 10 of our participants (see “Materials and methods” ***), as an indicator of the spatial spacing of the receptors array. Consistent with previous reports^46^, JND values (JND = 3.16 ± 0.98) varied substantially across our participants. Importantly, for each participant the three tested fingers had similar JND values (Table 1, last column). During their first session, the median tangential speeds of the participants were correlated with the mean JND measured across the three fingers (Supplementary material, Fig. 2B, *r* = 0.74, *p* = 0.014, *adjusted alpha* = 0.0056; 0.05/9). The correlations were high for the JNDs of the middle (Fig. 5A, *r* = 0.84, *p* = 0.0026; *adjusted alpha* = 0.0045; 0.05/11, *r*—95% confidence range [0.6,0.89], *p*—95% confidence range [0.0005,0.06], see “Materials and methods”) and index fingers and somewhat weaker for the ring finger (Supplementary material, Fig. 2A,C, Index finger: *r* = 0.725, *p* = 0.017, Ring finger: *r* = 0.43, *p* = 0.21, *adjusted alpha* = 0.0056; 0.05/9). Although participants could use any part of the three fingers array (we did not measure the movement of individual fingers), it is possible that the middle and index fingers were used more often than the ring finger, as previously reported in softness discrimination tasks^16,47^. A less frequent use may account for the weaker correlation of the ring finger. No significant dependency of participants median tangential speeds on the order or type of objects presented was found (*p* = 0.28, Kruskal–Wallis test).

**Table 1:** Two-point discrimination JND per participant: JND values in a static two-point discrimination task in the pads of the middle, index and ring fingers, and mean values across these fingers.

Participant	Index finger JND (mm)	Middle finger JND (mm)	Ring finger JND (mm)	Mean JND (mm)
AF	3	2	2	2.33 ± 0.57
EV	3	3	4	3.33 ± 0.57
MF	4	5	5	4.66 ± 0.57
AS	3	4	4	3.66 ± 0.57
YB	4	3.5	4	3.83 ± 0.28
SG	5	5	6	5.33 ± 0.57
DR	4	3	3.5	3.5 ± 0.5
HA	4	4	4	3.5 ± 0
MG	2	3	3	2.66 ± 0.57
ZA	3	2	3.5	2.83 ± 0.76

In order to assess the dependency of this correlation on the known unreliability of the two-point discrimination method (see “Discussion”), we have conducted a bootstrap test that covers, statistically, the estimated range of unreliability^48^ (see “Materials and methods”). The bootstrap analysis shows that the correlation coefficient (*r*) could rarely be < 0.5, and only in 8.1% of the cases one would get *p* > 0.05 (Fig. 5A, right). We thus assume that the finding of a positive significant correlation between the participants’ tangential speed and their mean JND is robust.

The temporal frequency of activation of adjacent mechanoreceptors equals the scanning speed divided by receptor spacing^45^. The positive correlation observed between the participants’ median speed and their JND may be consistent with an attempt to maintain this temporal frequency within a narrow range^49^. Indeed, in session I, the participant-specific mean frequencies ranged between 12.8 and 22.9 Hz (15.95 ± 3.18). The mean evaluated activation frequency and its range increased monotonically along consequent sessions (Middle-finger, Mean evaluated activation frequency: Ses1: 15.9 ± 3.18, Ses2: 17.07 ± 5.26, Ses3: 18.12 ± 4.9, Ses4: 18.14 ± 6.38, Range of activation frequencies: Ses1: 10.14, Ses2:19.2, Ses3: 15.45, Ses4: 22). The mechanoreceptor type that is most sensitive in the range of our evaluated activations frequencies in session I (between 12.8 and 22.9 Hz) is the rapidly adapting (RA)^43^. To test the potential effects of JND-dependent speed modulations on neuronal activations, we simulated the responses of grids of RA units with varying densities using the TouchSim computational model^50^ (see “Materials and methods”, Supplementary material, Fig. 2). The simulation shows that the scanning speeds, used by our participants in session I (between 34.4 and 77.14 mm/s) generate activation rates within a narrow range (Fig. 5B, range between vertical dashed lines). We compared the speed range used by our participants to different speed values using equal size windows (50 mm/s), across the simulation speed values. The window containing our participants speed values (30–80 mm/s) allowed for significantly higher spike rates than in other equal size windows (3.99 ± 0.008 spikes/s versus 3.73 ± 0.22 spikes/s, *p* = 0.004, Mann–Whitney U test). The mean variability between neighboring neurons was significantly lower in this window in comparison to the other windows (0.05 vs. 0.32 spikes/sec, *p* < 0.001, Mann–Whitney U test Fig. 5C).

### Palpation curvature inversely correlates with the participant’s idiosyncratic sensory adaptation time

One of the major differences induced by the *Linear* and *Oscillating* sub-classes of *CF* (Fig. 2B) was in the duration of continuous interactions between the fingertip and the shape’s edge. *Linear* motion induced longer epochs of such interactions than *Oscillating* motion.

A natural physiological feature that might be related to the choice between these strategies is the receptor adaptation rate. Receptors with slower adaptation processes allow longer epochs of similar stimulation and longer *Linear* motions. We have thus tested the effective adaptation times of our participants, while following edges of different line types (straight, tilted or curved) or following straight lines at different heights (see “Materials and methods”). Participants were asked to follow outlines, forward and backward, for 30 s. For each trial, the trajectory of the hand was analyzed and the time that elapsed from trial onset to the first deviation of the trajectory from the outline was considered as the adaptation time of that trial (Supplementary material, Fig. 2). The mean adaptation time of a participant (Τ_a_) was calculated across all her or his adaptation trials (see “Materials and methods”). In order to examine if curvier *Oscillating* motion indeed prolonged the effective adaptation time, we compared the adaptation times of our participants when following the shape’s outline using *Oscillating* and *Linear* motions (Experiment B). The adaptation times were longer with *Oscillating* motion for most of the participants (*Mdn *_*Oscillating*_ = 29.07 s, *IQR *_*Oscillating*_ = 30–26.3 s vs *Mdn *_*Linear*_ = 22.90 s, *IOR *_*Linear*_ = 29.8–17.3 s; *signed rank* = 9, *p* = 0.12, Wilcoxon signed-rank test; Fig. 5D). To estimate quantitatively the pattern of *CF*, we computed a curvature index for every trial (Fig. 5E). During the first session, the correlation between the participants’ Τ_a_ (22.29 ± 8.02, N = 10 participants) and their curvature index (0.26 ± 0.14) was high (Fig. 5F, r = − 0.77, *p* = 0.0095, *adjusted alpha* = 0.01; 0.05/5). Thus, naïve participants with shorter adaptation times used curvier movements, such that they increased the number of border crossings and shortened the epochs in which skin stimulations remained relatively constant. No significant dependency was found between the participants’ median curvature indices and the order or type of the presented items (*p* = 0.16, Kruskal–Wallis test).

To test the potential effects of *Linear* and *Oscillating* motion on neuronal activations, we simulated the responses of a grid of RA units to these motion types, using the TouchSim computational model^50^ (see “Materials and methods”). The simulation confirmed the prediction that employment of *Oscillating* motion induces more synchronous firing and an overall higher spike rate per neuron (*p* < 0.001, Mann–Whitney U test) than *Linear* motion (Fig. 5G). RA firing was triggered by both the onset and offset of tactile stimulation, simulating the indentation induced by contour scanning (see “Materials and methods”). While the intensity of RA firing could be controlled by their pressing force, the timing was controlled by the scanning trajectory—the curving of the scanning trajectory was adapted by our participants according to their adaptation times (Fig. 5F).

### The correlations between motion strategies and sensory thresholds diminish with practice

In the three sessions that followed the first session, the curvature index and the hand speed gradually lost their dependence on the participant’s Τ_a_ or JND (Fig. 5H, supplementary material, Fig. 2D). The decrease in correlation may result from transient, practice-induced changes in relevant physiological thresholds as previously reported^51^, from changes in strategy (for example, hand speed was shown previously to depend on the stimulus or the task^14,52,53^), or from both.

Practice-induced strategy changes are supported in our study by the changes in preference for *CF* versus *SC* palpation observed across sessions for a fraction of the participants, as well as by differences in visit-rate patterns that depended on their practice history (Supplementary material, Fig. 3).

## Discussion

This study describes the repertoire of hand movements strategies employed by human participants when required to recognize planar (2D) object shapes. Consistent with previous studies of 3D shapes^8,20,21^, we found that one major strategy was contour following *(CF)—*participants moved their fingers along the contours of the shape. In addition, we found a second major strategy—scanning (*SC*)—with which participants used large movements crossing the entire shape. Both strategies, and in particular *SC,* included focal scanning of specific object regions. *CF* was employed in two major patterns—*Linear* motion (moving along the contour) and *Oscillating* motion (moving back and forth across the contour while advancing along it). The participants’ movement trajectories were correlated across subsequent explorations of the same shape, such that the same locations on the shape were visited in similar relative times along the trajectory. This result is in line with the similarity in eye movements’ trajectories previously reported^34–37,54^. High-resolution tracking of hand movements revealed a strong dependency of critical movement parameters on two physiological thresholds— one spatial (JND) and one temporal (T_a_). When the participants were naïve to the task, their tangential velocities were correlated with their fingertip spatial resolution and their tendency to perform an *Oscillating* motion variant of *CF* was correlated with their mechanosensory adaptation time.

The correlations between scanning patterns and physiological thresholds gradually decreased with practice. One interpretation of these results is that, with practice, human participants adapt scanning strategies that compensate for their sensory limitations. Compensating strategy choice can be for example, using *SC* over *CF*. Such an adaptation can be beneficial for individuals with short T_a_ (this strategy was adopted by two of our participants, Supplementary Material, Fig. 3A). Another possible example for a compensating strategy choice can be the avoidance of shapes’ corners, of which exploration requires high spatial resolution. Such an adaptation may be beneficial for individuals with high JND values. Adaptations of hand movements strategies are consistent with previous reports^23,24,31,55^. The decrease in correlation may also result from practice-induced changes in relevant physiological thresholds as previously reported^51^ or from changes in both strategies and physiological thresholds.

Taken together, these results provide a detailed description of the specific hand movement strategies used for shape perception and initial information about the complex ways in which individual sensory abilities, practice repertoire and task-demands converge to an effective motor-sensory exploration strategy.

The main limitation of this study is the relatively small number of individual participants (n = 11). While the large sample size of high-resolution tracked trajectories (n = 1196 trajectories; total of 18,701s) allows the generalization of our findings to the typical case of palpation of planar objects by human subjects, the relatively small sample size of individual human subjects entails that the distribution of palpation patterns within groups of human subjects should be generalized with caution. For the same reason, the dependence of palpation parameters on physiological thresholds should be further studied, using additional physiological parameters and with larger groups of human subjects, before practically applied.

We attached the three palpating fingers (index, middle and ring) together, in order to be able to measure all motion components that are relevant to the perception of the objects. The participants were free to use any part of the affected sensory array, which was included the contact areas of the three fingertips. Thus, in a way, the three fingers were treated as one large finger. As far as it is known from the literature, tactile perception naturally involves coordinated motion of all contacting fingers: participants’ fingers were shown to have correlated positions and speeds in tactile search tasks^56^ as well as in a task designed to require only one finger^57^. Even when human participants were specifically instructed to use only one finger, low amplitude, correlated movements were seen in the other fingers^58^. Nevertheless, in the current study we did not examine the independent movement of each finger, a degree of freedom that might be treated differently by different participants.

Traditionally, two methods have been used to assess the spatial tactile resolution in specific skin areas of specific subjects: two points discrimination^59–61^ and grating orientation task^62^. Here we used the former method. In order to generalize our results across these methods we have computed our confidence levels based on the comparison between these two methods. Participants’ JND values were adjusted using values randomly picked from a distribution of differences between the two methods^48^. The adjusted JND values were compared to participants’ median speeds. The resulting r and p values adopted from these comparisons were used to estimate the confidence range of the relation between JND and speed (Fig. 5A, see “Materials and methods”).

In a pioneering study, Lederman and Klatzky termed the concept ‘Exploratory Procedures’ (EPs) to refer to the motor-sensory exploration strategies characterizing the palpation of various classes of 3D objects under specific tasks^8^. Here we zoomed into a one class of objects—planar shapes—and tried to characterize the EPs that are naturally employed by human participants when asked to identify shapes. Our results expand those of Lederman and Klatzky and their collaborators^8,20,21^ by showing that people employ not only *CF*, as observed by these researchers, but also *SC* motions, when exploring shape in 2D objects (Fig. 2). The SC motion resembles the ‘lateral motion’ EP, which was reported as the dominant strategy for texture palpation in 3D objects^8^.

Our high-resolution tracking system provided additional information about the nature of these strategies, revealing that human participants vary in the specific pattern of each EP—*Linear* and *Oscillating* motions for *CF* and uniform and non-uniform coverage of the shapes’ contours for both the *SC* and *CF*. Our tracking also revealed a frequent use of a palpation policy that was not describe before—focal palpation. Participants often dedicated a significant portion of time to explore specific regions of the objects. Focal palpation was more evident in *SC* trials, (Fig. 3) and depended on the properties of the explored object: objects with sharp angles were explored in a more focal manner. The tendency to use focal palpation was hardly affected by the participants’ physiological thresholds. Focal palpation was recently reported to frequently occur when blindfolded sighted human subjects explore objects (2D and 3D) using an active sensory substitution device^63^. Interestingly, such focal palpation was typically not observed with blind subjects exploring the same objects with the same device—their palpation covered the explored objects more uniformly. It thus seems that, in sighted subjects, focal palpation results from, or uses, the accumulated experience of perceiving similar objects visually.

Our high-resolution tracking was able to reveal large differences in participants’ hand movements. Using a similarity index, we were able to quantify the spatiotemporal similarity of the motion trajectories. We found that trajectories of the same participant at the exploration of the same shape were similar in a spatiotemporal manner. Such that the same locations on the shape were visited in the same relative time. The similarity between specific subjects-shape pairs was higher than similarity of different subjects exploring the same shape, or the same subject exploring different shapes (Fig. 4). The similarity in subject trajectories, is in line with the repetition of eye movements’ trajectories in subsequent views of the same picture^33–37^. Like in the case of eye movements, initial hand movements exploration were more similar than final ones^38,39^. The high similarity between the trajectories of the same subject exploring the same shape suggests that hand movements adapt to both the explored object as previously suggested^8^ and to an additional idiosyncratic factor.

One idiosyncratic factor may be physiological thresholds. Sensory-motor behavior depends on the physiological parameters of sensory receptors^64,65^. Consistently, our results clearly demonstrate that the choice of motion strategy depends strongly on sensory physiology. The speed of motion decreased with increased spatial resolution (reduced JND) at the fingertip, and the degree of motion curvature increased with faster adaptation times (T_a_, Fig. 5). These correlations were very strong when our participants were naïve to the task: during session I, the physiological measures, JND and T_a_, could explain 70% and 59% of the variability in the tangential speed and curvature index, respectively. Such behavior is expected when the tactile system is concerned with maintaining specific sensory variables in their ‘working ranges’, i.e., ranges that allow satisfactory perception^25,42,66^. Thus, rats maintain head azimuth and whisker speed^29^ and humans maintain hand coordination and hand speed when localizing objects around them^17^ and ocular drift speed when viewing simple 2D shapes^67^.

When scanning planar objects via touch, humans also often attempt to maintain temporal activation variables within certain ranges. Thus, humans, when perceiving textures, reduce hand speeds with higher external spatial frequencies, such as to maintain the temporal frequencies within a limited range^14^. When exploring surfaces with different geometry and friction attributes, they modify radial and tangential forces, together with lateral hand speeds, such as to maintain a certain amount of skin deformations^30^. Maintaining such sensory signals within certain ranges is expected to facilitate their predictive processing within brain circuits^25,68–70^. Variables that are actively maintained within specific ranges are termed ‘controlled variables’^66,71–73^. Preserving them in preferred working ranges requires a closed-loop architecture, in which the variables can be sensed and manipulated. As these controlled variables are serving perception, their control is likely to optimize sensation^11,71,74–76^.

Controlling hand speed in the current experiment resulted in maintaining the mean temporal frequency of fingertip activations (when crossing contour edges) between ~ 10 and 40 Hz across participants and sessions. In texture-related tasks the effective temporal frequency of individual receptor activation is typically maintained between 15 and 30 Hz^14^. This frequency range is optimal for activating rapidly-adapting (RA) receptors at the primate fingertip^42–44^. Emphasis of RA receptors in this experiment is in line with the fact that the height of our shapes was 25 microns, which is better sensed by RA receptors compared with slowly-adapting (SA) receptors^43,44,64^.

Our simulations further suggested that the JND-dependent control of scanning speed observed here (Fig. 5A) resulted in a uniform activation rate of RA receptors (Fig. 5B,C). Another support for the conjecture that the tactile system attempts to maintain uniform activation rates comes from our behavioral adaptation measurements. These results suggest that our participants made an effort to prevent receptor adaptation—participants with shorter adaptation times used curvier movements (Fig. 5F), which were likely to reduce receptor adaptation and thus maintain activation levels (Fig. 5G). These results suggest the response magnitude is also a controlled variable in planar shape perception. This suggests that the processing of sensory data in such cases assume a uniform response magnitude across neighboring receptors, presumably for allowing simple comparisons within and across the relevant receptor sheets.

Our high-resolution tracking of hand movements during planar shape perception revealed specific palpation strategies and idiosyncrasy in hand movement patterning, in a way that was specific to subject-object pairs. Two physiological thresholds—a spatial (JND) one and a temporal (T_a_) one, could partially account for the variability in movement pattering when participants were naïve to the task. These results suggest that hand movements adapt in an idiosyncratic manner to both the tactile object and to idiosyncratic physiological thresholds.

These results may have applicative value. Previously we have shown that people can be trapped in a wrong strategy, from which they can be trained out using appropriate guidance^14^. Our current findings indicate that specific strategies can be fitted to specific individuals based on their idiosyncratic physiological thresholds. Thus, teaching of complicated tactile skills, such as Braille reading, can be significantly facilitated by guiding individuals to use scanning patterns that fit their idiosyncratic thresholds.

## Materials and methods

### Overview of experimental design

This study was composed of two experiments. The first experiment (Experiment A) was designed to study the characteristics of tactile scanning when perceiving planar shapes. In the second experiment (Experiment B, conducted 18 months after Experiment A), adaptation times and spatial resolutions of most (10 out of 11) of the participants who took part in Experiment A were measured. The experimental procedures were approved by the Helsinki committee of the Tel Aviv Sourasky Medical Center and the Weizmann Institute Review Board (IRB).

### Experiment A: Shape recognition

#### Participants

Eleven right-handed participants [seven female and four male students, aged 21–32, (25.36 ± 3.17) years] took part in the study. Informed consent was obtained from all subjects for both study participation and publication of identifying information or images in an online open-access publication, in accordance with the approved declaration of Helsinki or the Weizmann Institute Review Board (IRB).The participants were paid for their participation. Participants had no previous experience with tactile recognition tasks.

#### Sample size

Sample size in this experiment is similar to the sample size used in previous works which aimed to characterize trajectories of hand motion ^30,63,77–79^. This sample (11 subjects recorded for four or five sessions, total of 1196 trials across all subjects and sessions) allowed the characterization of hand movement strategies and the study of their dependencies on the sensed shapes and on the measured physiological thresholds. Overall, 26 statistical tests were conducted in this study, 61.53% of the tests indicating significant differences, and 10% of those yielding insignificant results, had statistical power > 0.8.

#### Tactile shapes

Objects were engraved on aluminum boards of size 150 × 150 mm, such that the area inside the shape was raised to 25 μm in relation to the board surrounding. Shapes were divided into three sets: Objects—sets A and B (Set A: triangle, circle, rectangle, square, parallelogram; Fig. 1B, black. Set B: ellipse, hexagon, L, trapezoid, diamond; Fig. 1B, blue) and a Features set (Fig. 1B, gray). The Features set included three blocks: ‘Angle’ block (ninety, acute or obtuse angles), ‘Tilt’ block (tilt-right, tilt-left or straight vertical line) and ‘Curvature’ block (concave, convex or straight vertical line).

#### Experiment design and procedure

Participants were asked to identify two-dimensional (2D) engraved objects or features (Fig. 1B). Before the beginning of the first session, participants saw illustrations of all the objects or features that were presented in that session, and they were shortly trained by palpating on one of the objects or features until they reported that they understood the task. The training was short, and its main purpose was to allow participants to distinguish between the board surface and the surface of the tactile shape. No effect of this short training on the presented results was found. Participants performed four or five experimental sessions. Each session lasted approximately 45 min and was conducted on a different day. The longest experiment period lasted 15 days. The order of object presentation within each session was determined randomly, within one out of three possible presentation protocols (Supplementary material, Fig. 5). Whenever new shapes were introduced, a visual illustration of them was presented to participants at the start of the relevant session. At the beginning of each trial, the participant’s gloved-hand was placed (Fig. 1A) a few centimeters above the center of the shape or feature board, and the trial began when the participant was allowed to put their hand on the shape (see “Testing apparatus” below). Participants were requested to raise their palpating hand and name the shape placed in front of them when they identified it, as well as to report their confidence level. Trials ended with the participant's declaration or after a time limit of 30 s (seconds) (whichever came first). A feedback was given on whether the answer was correct or not. In each session or block (see below), the order of trials was randomized, if the random order of presentation did not contain all the shapes that should have been included in the session, a new random order was generated such that each object was presented at least once. Trial presentation order within a session or block was kept constant across participants. In order to generalize over different tactile items and over differences in idiosyncratic experiences, three presentation protocols were used. In the first, only objects were presented. In the second, isolated tactile features were presented before full objects were presented. In the third, novel objects were presented in each session. Each protocol entailed a specific forced-choice report pattern, which emerged from the combination of tactile items presented. These variations preclude dependency of the results on a specific presentation order, specific tactile experience or specific forced-choice reporting pattern. Presentation protocols: (Supplementary material, Fig. 5).

##### Session protocol I—objects

During sessions I–III (20 trials each), five participants were presented with a fixed set of five geometrical objects (Fig. 1B, Set A, black). The order of trials was random but kept constant between participants. During session IV (34 trials) they were presented with novel geometrical objects (Fig. 1B, Set B, blue). These objects that were not included in the previous sessions. In each session they performed a five-alternative forced choice (5-AFC) recognition task.

##### Session protocol II—features

During sessions I–III (30 trials each), three participants were presented with a set of geometrical features (Fig. 1B, gray). Features were presented in three blocks, 10 trials each: ‘Angle’ block, ‘Tilt’ block or ‘Curvature’ block. Before each block, participants were notified which block is presented, and they were requested to name the presented feature in a three-alternative forced choice (3-AFC) recognition task. The order of blocks was randomized between participants and kept constant for each participant in all three sessions. The order of trials within each block was randomized but kept constant between participants. During session IV (34 trials) they were presented with novel geometrical objects (Fig. 1B, Set B, blue).

##### Session protocol III—non fixed objects

During sessions I–V (35 trials each), three participants were presented with a non-fixed set of geometrical objects. During session I, the participants were presented with a set of five objects and during sessions II–V, one or two objects were replaced by novel ones (Supplementary material, Fig. 5, right, yellow). A visual illustration of the novel shapes were presented at the beginning of the session. The task gradually changed from a 5-AFC (session I) to a 9-AFC recognition task (session V; only five objects were presented in each session).

#### Hand tracking

Hand motion was tracked in 3D coordinates (x, y, z), using Vicon 612 motion capture system (Vicon Motion Systems Ltd, Oxford,.UK) and the Nexus 2.5 software. A custom ‘Vicon labeling Skeleton Template’ (VST) of the hand was designed. Three segments and four markers attached above the wrist carpal bones and the middle finger’s middle and proximal phalanges were included in the VST (Fig. 1A). At the beginning of each session, the VST was calibrated for the current subject’s parameters, and a labeling skeleton file (VSK) was created. Hand motion was sampled at 200 Hz (76.5% of trials), 100 Hz (5.7%) and 240 Hz (17.8%), (see “Vicon tracking” within “Data analysis” below).

#### Testing apparatus

Experiment took place in a Vicon arena. Participants sat in front of a table on which the stimulus was placed. The aluminum board was placed in a plastic frame, preventing its movement. Participants were blindfolded and wore a glove on their right hand. Four Vicon designated markers were connected to the glove, in a way that two markers were connected above the middle and proximal phalanges of the middle finger and two above the wrist carpal bones (Fig. 1A). In order to simplify and standardize the experiment, degrees of freedom were reduces by banding together the index, middle and ring fingers with a tape—as if all three fingers belong to one surface plane. This restriction facilitated analysis and allowed comparison to other existing datasets. Three corresponding fingertips of the glove were cut, such that the finger pads were uncovered. Two glove sizes were used and chosen according to participant’s hand size.

#### Data analysis

##### Vicon tracking

Trajectories of one marker were analyzed—the ‘tip’ marker that was placed above the middle finger, middle phalanges (closest to the finger pad, Fig. 1A). In a fraction of the sessions, the number of performed trials was smaller than planned (10 out of 47 sessions, 21.27%). A fraction of performed trials was excluded due to missing capture frames; the analysis throughout the paper includes the remaining number of trials (1196 out of 1367 trials, 87.4%). A fraction of these trials was not sampled at 200 Hz but at 100 Hz (5.7%) and 240 Hz (17.8%). These trials were resampled to 200 Hz: 100 Hz trials were linearly interpolated by a factor of two using the MATLAB function ‘interp1’; 240 Hz trials were linearly interpolated by a factor of five using the ‘interp1’ MATLAB function and then down sampled by a factor of six using the ‘downsample’ MATLAB function. In some sessions (n = 22 out of 47 sessions, 46.8%), the calibration of hand position in relation to the shape was lost. To compensate for differences in calibration quality, for each session one trial with clear hand-trajectory orientation relative to the borderlines of the shape was chosen and the translation and rotation between this trajectory and the shape were calculated. These parameters were used to shift and rotate all the hand trajectories in that session.

##### Trial start, end and reaction time

At the beginning and end of each trial, participants lowered or raised their hands, respectively (see experimental procedure). The point of hand lowering was marked as the first frame in which the height in the z-axis was equal or smaller than the median z height in the rest of the trial. The point of hand raising was marked in a similar way. Hand velocities in the z-axis were smoothed using a moving average (MATLAB function, ‘movmean’, window size = 30 samples). A histogram of all data was plotted, forming a bi-modal distribution. Hand raising was marked as the first frame in which z-speed was higher than a threshold marking the second mode. In addition, in order to exclude 2D movements that accompanied hand lowering or raising, trial start (t = 0) was defined as the first frame after hand lowering in which the hand was at < 5 mm from the shape outline. Trial end was marked as the last frame for which all consequent frames were > 5 mm from the contour. Trial reaction time (RT) was the difference between trial start and trial end.

##### Classification of movement types

Following initial screening of trial trajectories, we aimed at classifying the trials into two types: contour following (*CF)* trials were defined as trials in which the hand remained in the vicinity of the object outline. Scanning (*SC*) trials were defined as trials in which the trajectory crossed between distant parts of the objects’ contour. Trials were classified according to this distinction using two methods: Algorithmic and perceptual (by human observers). The analysis throughout the paper is based on the algorithmic classification. The human observer classification was used as a verification method. The analyses based on it are presented as supplementary material (Supplementary material, Fig. 4).

##### Algorithmic classification

The algorithm used heuristic criteria that were based on our initial inspections of motion strategies. For each original shape two similar ‘guiding shapes’ were plotted around its centroid: an inner and outer guiding shapes whose areas were 0.25 and 1.5 of the original shape, respectively. The 'L' and 'convex' objects were exceptional (see Supplementary material, Fig. 4). Scanning trials (*SC*) were trials in which either the hand crossed the inner guiding shape (Fig. 2A, red, right top row), or those in which the hand did not remain between the inner and outer guiding shapes for more than 75% of the time (Fig. 2A, red, left, bottom row), or both (Fig. 2A, right, red, bottom row). *CF* trials (Fig. 2A, blue, top row) were trials in which the hand remained in the buffer between the inner and outer guiding shapes for more than 75% of the time and did not cross the inner guiding shape.

##### Human observer classification

Trials were classified by two human observers—one of the authors (NM) and a naïve observer (SM). *CF* trajectories were defined as those in which the shape center was not crossed, and the trajectory was close to the contour. *SC* trials were those in which the trajectory crossed the shape center and included movements from one object’s sides to another. NM followed these rules. SM was shown one example of *SC* and one of *CF* trajectories. In case it was not possible to determine if a trajectory belongs to the one of the *CF* or *SC* categories the trajectory was classified as ‘*Other*’. Each observer classified all trials twice consecutively, and the second set of labeling was used for analysis. The two observers differed in the percentage of labeling trials as ‘*Other*’ (NM, 11.8%; SM, 15.6%) and agreed on 66.6% of the trials (*a*_Krippendorff’s_ = 0.44). When removing all trials that were classified as ‘*Other’* by either observer, and examining only *CF* or *SC* trials, observers agreed on 78% of the trials (*a*_Krippendorff’s_ = 0.66). Only trials that were equally labeled by both observers as *CF* or *SC* trials were used for the verification of the algorithm categorization (Supplementary material, Fig. 4).

#### Focal index

To evaluate the distribution of palpation density along the shapes’ outlines, a focal index was used. Circles at a radius of 10 mm were plotted on the object outline, such that the distance between their centers was 0.5 mm. The traveled hand-trajectory distance in each circle was computed. As a measure of dispersion, the difference in traveled distance between the most visited (Fig. 3A, left, pink circle) and least visited (Fig. 3A, left, green circle) areas was computed (Fig. 3A, right) and divided by their sum.

$$Focal\, index=\frac{\mathrm{most\, visited}-\mathrm{least\, visited}}{\mathrm{most\, visited}+\mathrm{least\, visited}}$$

The least-visited circle was determined after removing circles that were not visited or circles that overlapped with non-visited circles.

#### Similarity index

To quantify the spatiotemporal similarity of the motion trajectories in different trials a similarity index was calculated for pairs of trials. For each pair, the trajectory of the longer trial was down-sampled to match the length of the shorter trial, using the MATLAB function ‘resample’. Then, Pearson r was calculated separately for the horizontal (r_x_) and vertical (r_y_) components of the two trajectories (Fig. 4A), and averaged:

$$Similarity\, index=\frac{{\mathrm{r}}_{x}+{\mathrm{r}}_{y}}{2}$$

The similarity index was computed for pairs of trajectories of the same subject exploring the same object (n = 7383 pairs, for all subjects and shapes), the same subject exploring different shapes (n = 61,004 pairs), and different subjects exploring the same shape (n = 41,700 pairs). In order to evaluate a null distribution of the similarity index (Fig. 4B), a normal distribution containing the possible values of the similarity index ($\pm$ 1) was created. The distribution had a mean zero and its $\pm$ 2.58 SD were equal to $\pm$ 1, respectively.

#### Trajectory curvature

Curvature evaluation was calculated in the following way: each trial’s hand trace was smoothed using a moving average with a window size of three samples. The trace was divided into segments of 20 mm length; the overlap between consequent segments was 0.01% (0.2 mm). Curvature index was defined as the subtraction of the shortest distance between the beginning and end of the segment’s coordinates from the segment-traveled distance, divided by the traveled distance:

$$K\, segment=\frac{traveld\, distance-shortest\, distance}{traveld\, distance}$$

The value of the curvature index is in the range between 0 and 1: The index is closer to zero as segment trace resembles a straight line (Fig. 5E, dark blue) and to one as it is more curved (Fig. 5E, dark red). The median of the indices of all segments in a trial was assigned as the trial curvature index. The constant-length segmentation was preferred over the constant-time segmentation because stronger curvatures are usually accompanied by slower movement^80,81^, which would lead to over-estimation of curved movements in the latter case.

#### Trial entropy

In order to evaluate trajectories entropy, a gray scale image of each trial trajectory was created using the MATLAB function ‘hist3’. The entire stimulus board (150 × 150 mm) was included. Bins with the size of 2 × 2 mm were used. The entropy of this grayscale image was calculated using the MATLAB function ‘entropy’.

#### Visit rates

The number of visits of the hand’s trajectory in each 2 × 2 mm bin were counted and divided by trial duration, and smoothed using the MATLAB function ‘fspecial’ with a averaging filter in the size of 2 × 2 mm.

### Experiment B: Spatial and temporal thresholds

In this experiment, the spatial resolution and effective adaptation time profiles of 10 of our participants were tested. The experiment was conducted 18 months after experiment A. Experiment aim was to test the possible differences between participants’ thresholds and examine if these differences could account for idiosyncrasy in *CF* variants (Fig. 2B).

#### Participants

Ten participants who participated in experiment-A took part in this experiment. The participants were naïve to the purpose of the experiment and were paid for their participation. Informed consent was obtained from all subjects for both study participation and publication of identifying information or images in an online open-access publication, in accordance with the approved declaration of Helsinki or the Weizmann Institute Review Board (IRB).

### Task 1: Temporal profile of sensory adaptation

#### Motion tracking

Trials were filmed (sampling frequency—30 Hz) and were later analyzed using the MATLAB vision toolbox and the ‘tracker’ function.

#### Tactile objects

The ‘straight’, ‘right tilted’ and ‘convex’ outlines from the features set were used (Fig. 1B, right, gray). In addition, eight identical-size rectangles raised to different heights (engraved on an aluminum board) were used. Rectangle heights ranged from 10 to 80 µm and differed from one another by 10 µm.

#### Testing apparatus

Apparatus was identical to the apparatus in Experiment A, apart from the fact that it did not take place in the Vicon arena. The same glove was used, which had one polyester marker connected above the middle phalanges of the middle finger.

#### Design and procedure

This experiment aimed to test the temporal profile of sensory adaptation while following the contour of our tactile objects. The participants were requested to use two types of motions: *Linear* and *Oscillating* (Fig. 2B). The experiment included one session. Session duration was approximately one hour. At the beginning of the session, these motions were demonstrated by the experimenter and then practiced by the participants, first on a desk and later using the ‘rectangle’ stimulus board (Fig. 1B, Set A, black). Before each trial, participants were instructed which motion type they should use. After each trial, a break of 45 s was taken, in which participant’s hand was placed such that the finger pads were in the air. This was meant to allow full recovery of both slowly and rapidly adapting receptors^82^. Before each trial, the participant’s hand was placed on the starting point of different outlines, and they were asked to report if they could feel the contour. Participants were instructed to follow the contour using *Oscillating* or *Linear* motion, until they were told by the experimenter that the trial ended. Trial duration was 30 s. During each trial, the contour was tracked either once or more (moving back and forth along it), depending on hand velocity. The participants were not asked to report anything nor were they given any feedback.

#### Stimuli types: two tactile arrays were used

##### Array with different outline types

The outline was either ‘straight’, ‘right tilted’ or a ‘convex' engraved shapes raised to 25 μm (Fig. 1B, Features set, gray). This task included six trials, as each outline type was followed using both motion types (*Oscillating* and *Linear*). The order of the trials was random and kept constant between participants.

##### Array with different outline heights

A board with 8 rectangles was used. Rectangle lines heights ranged from 10 to 80 µm and differed from one another by 10 µm. The task included 16 trials, as each outline height was followed using both motion types. The task was performed in two blocks—*Oscillating* and *Linear*. The order of blocks was random and kept constant across participants. The order of trials within each block was randomized but kept constant across participants.

### Data analysis

For each trial we identified the first time in which the tracking hand lost the outline using visual inspection of the trial’s movies. Two human observers examined the movies—one of the authors (NM) and a naïve observer (SG). Each observer marked the first point in which the hand’s trajectory clearly deviated from the outline and assigned the time duration between trial start and the point of deviation as the trial τ_a_ (Supplementary material, Fig. 2F, left and second left). In trials in which participants did not deviate from the outline the observers assigned τ_a_ as the maximal trial duration (30 s, Supplementary material, Fig. 2F, right and second right). Trials in which it was not clear whether participants indeed lost the outline were excluded. For the majority of trials (n = 170 out of 225, 75.56%) both observers confidently assigned a τ_a_. For these trials, the correlations between the τ_a_s assigned by the two observers, for *Oscillating* and *Linear* motion trials, were *r* = 0.73 and *r* = 0.68, respectively (*p* < 0.005, Supplementary material, Fig. 2G, left and middle). The distribution of the differences between the two observers’ τ_a_s exhibited a clear mode at 0 and a secondary mode between 0 and 5 s (Supplementary material, Fig. 2G, right). Only trials for which the difference was < 5 s, which composed 86.47% (147 out of 170) of the trials, were included in this analysis. For these trials, the τ_a_ was taken as the mean of both observers’ τ_a_s_._

### Task 2: Spatial resolution

The spatial just-noticeable difference (JND) of each participant was measured using a static two-point discrimination test, applied to the pads of index, middle and ring fingers, similar to a procedure previously described^60^. Participants placed their hand comfortably on a table and were blindfolded. Participants were asked to report whether they feel contact in one or two points on their skin. The task was demonstrated on the participants’ forearm before starting the experiment. An adjustable compass was used. The interval between the two tips of the compass was gradually reduced until the participant could not differentiate between the two points. An effort was made by the experimenter to keep the same amount of pressure. Threshold was determined as the first interval at which the two points could not be distinguished. The order of measured fingers was ring, middle and index finger for all participants.

#### Assessment of the participants’ spatial tactile resolution

The reliability of the two points discrimination method in assessing spatial tactile resolution had been debated^62,83^. Therefore, the dependency of the correlation of our participants’ speed with their spatial tactile resolution (as shown in Fig. 5A) on the method used to assess the latter was examined. Two such methods were considered—our JND assessment and a grating orientation task^62^. The limits of agreement between two-points discrimination and grating orientation task thresholds (*Mean *_*methods difference*_ = 0 mm, *95% limits of agreement* =  ± 1 mm^48^) were used for a bootstrap testing. At each iteration (n = 1000), each participant’s JND was added a value randomly picked from the distribution of differences between the two methods. Correlation was tested between speed and adjusted JNDs values. The thousand iterations allowed us to assess the confidence range (Fig. 5A) for the correlation of participants’ speed with their spatial tactile resolution.

##### Statistical analysis—experiments A and B

Unless stated otherwise, the compared distributions were tested for normality using the Anderson–Darling test. If at least one of the compared distributions was recognized as non-normal, the Mann–Whitney U test (non-paired comparisons, two tailed), or Wilcoxon signed-rank test (paired comparisons, two tailed) were used. Otherwise, a two-tailed (independent or paired) t-test was used. Categorical data was tested using the Chi-square test of independence using all of the trials in each protocol session (84–146 trials). Multiple comparisons were corrected using the Bonferroni method. Since in some of the compared distribution part of the observations were dependent (trials belonging to the same subject), significance was additionally tested using bootstrap. Unless stated otherwise, all significance reports are based on both a model-based (one of the aforementioned) and a bootstrap test.

Bootstrap was performed in the following way: trials from both of the compared groups were mixed to one pool. Ten thousand iterations were used. At each iteration, two samples were taken from the mixed pool. One sample was at the size of the number of trials of comparison group I and the second at the size of the number of trials of group II. A difference calculation was repeated for each iteration. The fraction of bootstrap values that were more extreme (larger or smaller, depending on the case) than the experimental one was reported as the boundary of the probability of getting the experimental value by chance. In order to verify that the three sessions protocols were not different in terms of their kinematics, median values of compared kinematics (tangential speed, focal, curvature and similarity indices) were examined using the Kruskal–Wallis test.

Inter-rater reliability was evaluated using Krippendorff’s Alpha. Post-hoc power analysis was performed using the GPower software. For each result, the effect size (r value in the case of correlation, chosen’s d in the case of Mann–Whitney U test and Wilcoxon signed- rank test, epsilon-squared in the case of the Kruskal–Wallis test), alpha level and the sample size (the number of subjects, trajectories, trajectories pairs or simulated neurons) were fed into the GPower software. The adjusted alpha level was used in the case of multiple comparisons which were corrected using the Bonferroni method. Default procedures for power calculation of GPower were used: the asymptotic relative efficiency method for the calculation of power for Mann–Whitney U test and Wilcoxon signed- rank test and the exact distributions method for the calculation of power in the case of Pearson’s correlations. Post-hoc power of the Kruskal–Wallis test was performed using the R function ‘kwpower’. When calculating the power of results that their significance was assessed using Mann–Whitney U, Wilcoxon signed- rank test or Kruskal–Wallis test distributions shape was evaluated as logistic.

### Simulations

#### Relation between speed and spatial resolution

To test the effects of JND-dependent speed modulations on neuronal activations, simulations of mechanoreceptor responses were performed using TouchSim^50^. In order to simulate fingers with different receptors spacing, five grids of rapidly-adapting (RA) mechanoreceptive units were formed. These grids varied in the inter-receptor distances (2–4 mm, with 0.5 mm intervals). The resulting grid sizes were in the size of 10.5 × 10.5 and 12.5 × 12.5 mm (Supplementary material, Fig. 2E, left). Grids were created using the TouchSim built-in function ‘affpop grid’. JND values larger than 4 mm created grids with smaller number of neurons (9 versus 16–49 neurons) and smaller dimensions (9 × 9 and 10 × 10 mm) and were therefore not used. The stimulus was comprised of two columns of pins, aligned between the two left-most receptor columns (Supplementary material, Fig. 2E, right). The pin radius was 0.5 mm. In order to simulate different scanning speeds, in each simulation run, the two stimulus columns were pressed onto the finger grid with a delay that corresponded to the simulated scanning speed. Scanning speed was varied between 20 and 250 mm/s (with intervals of 10 mm/s) and pressing duration was 0.2 s. The duration of each simulation was 1 s, and the sampling rate was 5000 Hz. Default parameters of the model were used to determine the stimulus indentation depth: indentation was implemented as a sine wave with an amplitude varying between 0.5 and 1.5 mm. The resulting spike counts per each grid in all tested speed values were smoothed (moving average, window size = 5 samples).

#### Differences between *Linear* and *Oscillating CF*

The effect of *Linear* and *Oscillating CF* (Fig. 2B) on receptors activations was tested. A grid of RA units with 1 mm receptors spacing and in the size of 12.5 × 12.5 mm was formed (Supplementary material, Fig. 2E, left). The stimulus was comprised of one column of pins, aligned between the two left-most receptor columns (Supplementary material, Fig. 2E, right). Pin radius was 0.5 mm. In order to simulate *Linear CF*, the pins were pressed one after another. To simulate *Oscillating CF*, every 0.25 s, pins pressing was stopped for 0.2 s. The stop in pin pressing aimed to mimic the deviation from the contour. Default parameters of the model were used to determine the stimulus indentation depth: indentation was implemented as a sine wave with an amplitude varying between 0.5 and 1.5 mm. The duration of the simulation was 2 s and the sampling rate was 5000 Hz.

## Supplementary Information

Supplementary Information.
