# Supplementary material for: Idiosyncratic selection of active touch for shape perception
Source: Sci Rep. 2022 Feb 21;12:2922. doi: 10.1038/s41598-022-06807-2 (PMC8861104; doi:10.1038/s41598-022-06807-2)
Supplement: Supplementary file 1 — Supplementary Information. [file 41598_2022_6807_MOESM1_ESM.pdf]

Title: Idiosyncratic selection of active touch for shape perception

Abbreviated title: Active touch strategies underlying shape perception.

Authors names and affiliations: Neomi Mizrachi ^ #, Guy Nelinger #, Ehud Ahissar ^ # *, Amos Arieli ^ # *

Neurobiology department, Weizmann Institute of Science, 7610001 Rehovot, Israel

* Equal contribution

^ Corresponding author.

Corresponding authors email addresses: Ehud Ahissar ehud.ahissar@weizmann.ac.il Amos Arieli amos.arieli@weizmann.ac.il Neomi Mizrachi neomi.mizrachi@weizmann.ac.il

SUPPLEMENTARY MATERIAL

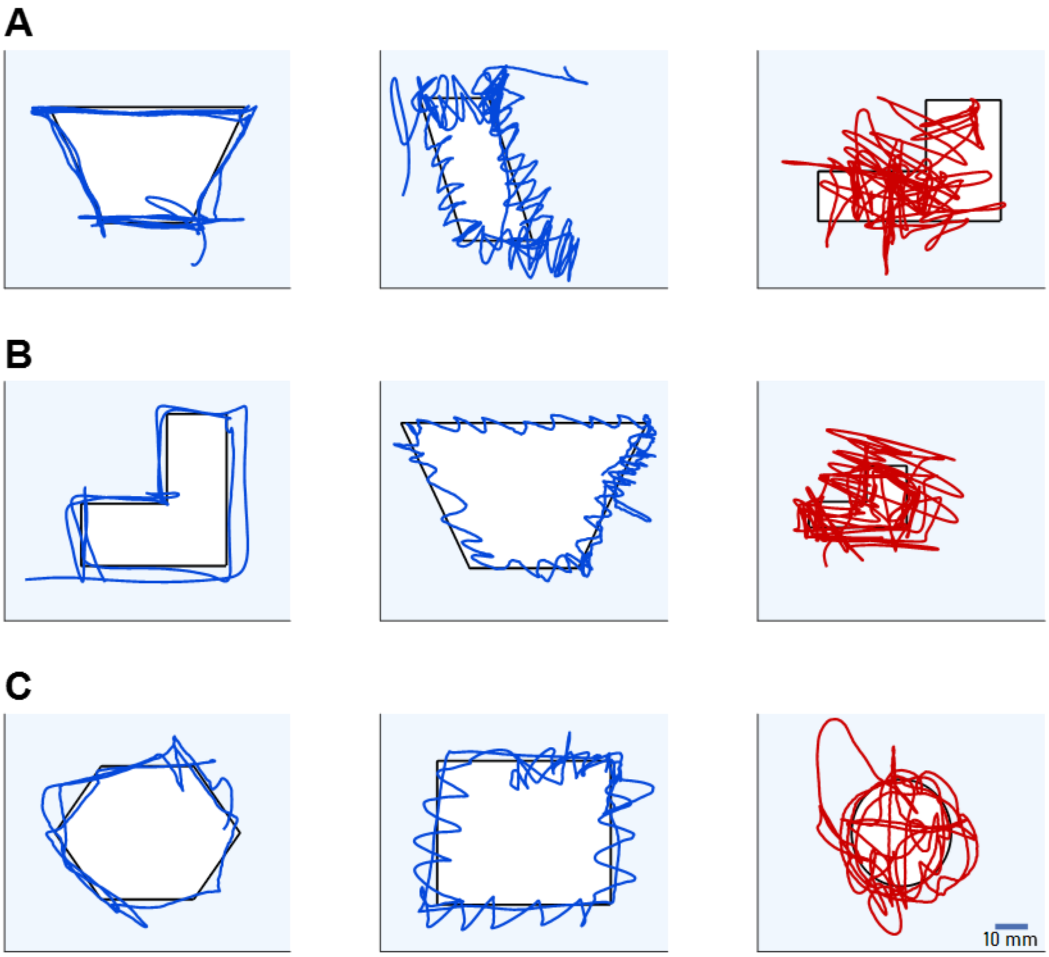

Supplementary Figure 1. Example of *CF* and *SC* trials for each session protocol**.** Examples of *Linear* and *Oscillating CF* trials (blue, left and middle columns, corresponding) and *SC* trials (red, right column) for session protocol I (A), II (B) or III (C). Scale bar (C, right) stands for all example trials.

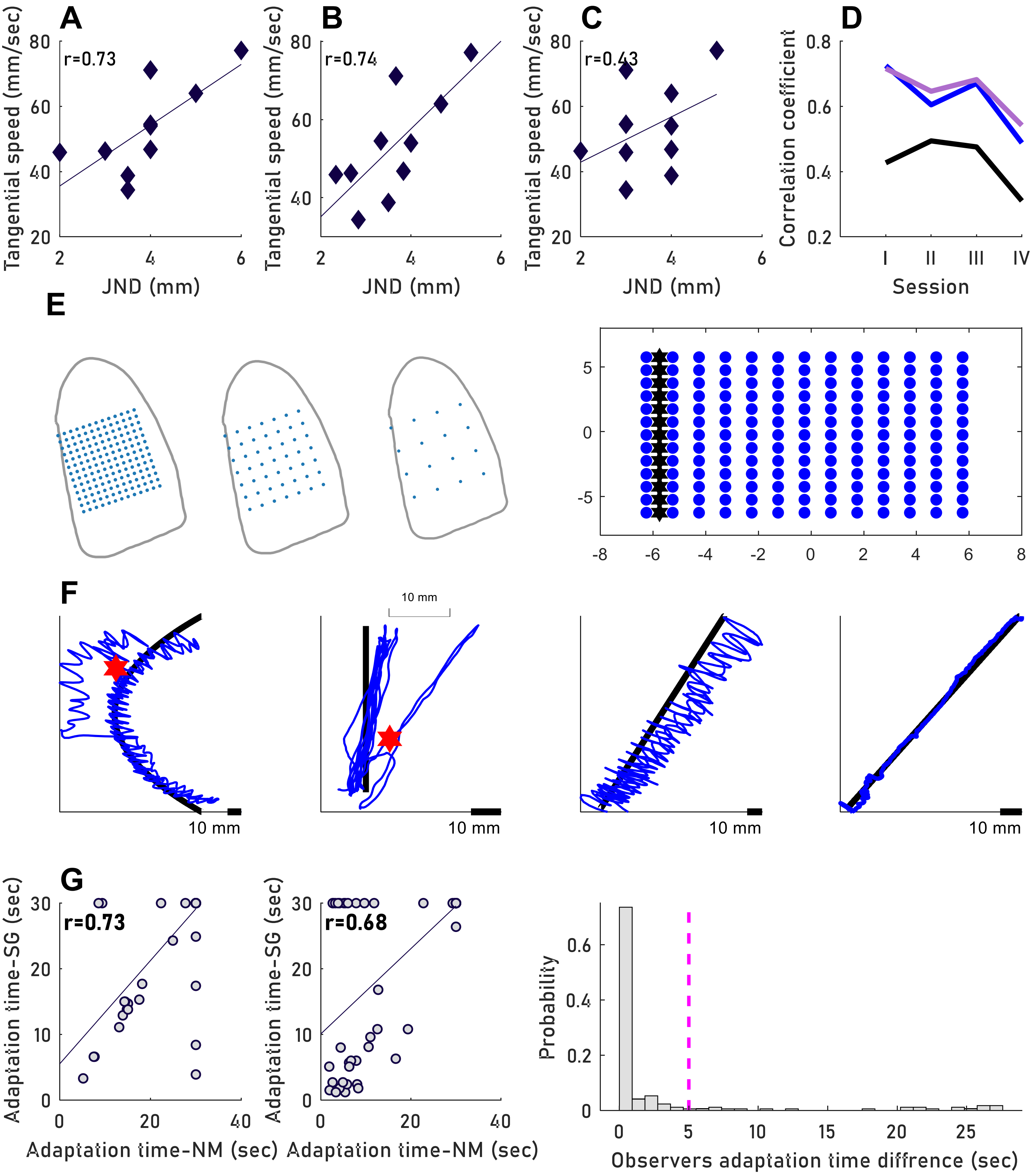

Supplementary Figure 2. Dependency on spatial resolution in different fingers (A, B, C) Each data point represent one participant's tangential speed (y-axis) across all trials of session one and its JND value (x-axis) at the index (A), mean JND value of the three fingers (B) or ring finger (C). (D) Correlation coefficient between JND and tangential speed (y-axis) per session (x-axis) for either the index finger (blue), ring finger (black), or the mean JND value of all fingers (purple). (E) Simulations: Left: An example of three finger grids, with either 1 mm (left), 2 mm (middle) or 4 mm (right) distance between units. Right: Stimulus columns (black) were placed between the units' columns (blue). In the presented grid the distance between units is 1 mm. (F) Trials 'adaptation time: Hand trajectory of outline-following using *Oscillating* or *Linear* motion. In the case there was a deviation, the point of deviation from the outline is marked (red pentagram). The elapsed time from trial start to the deviation point is the trial τ_a. In the case of no deviation from the outline (right and second right trial) τ_a was assigned as the maximal trial duration (30 sec). (G) Each data point represents the trial adaptation time marked by the two observers (x-axis-NM, y-axis-SG), for *Oscillating* trials (left) or *Linear* trials (middle), p<0.005 for both conditions. Right-Histogram of the differences between observer's adaptation times in absolute value. Trials in which the difference was bigger than 5 sec (pink dashed line) were excluded.

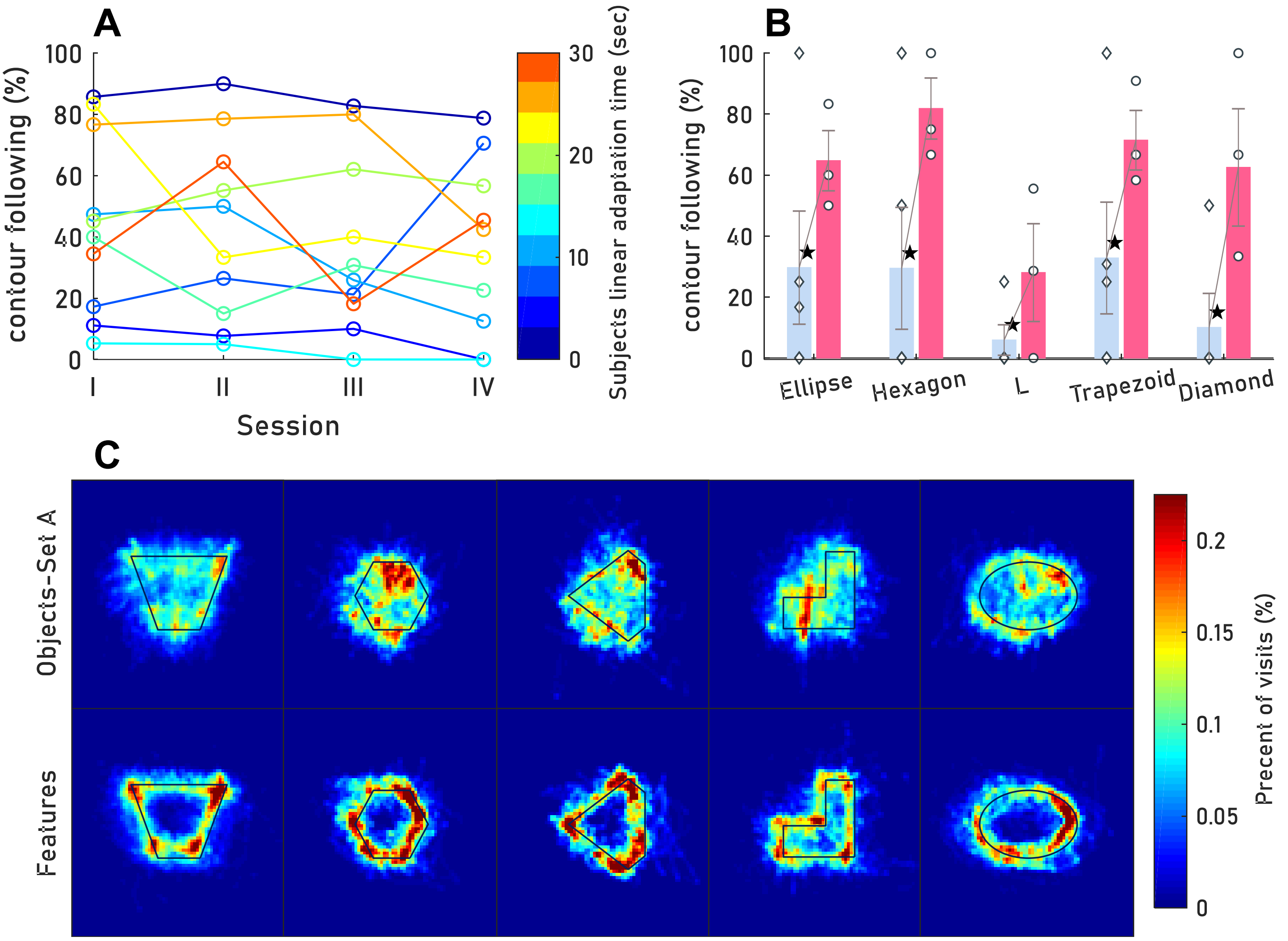

Supplementary Figure 3. Effect of practice on strategy choice. (A) Each line represents the percent of *CF* (y-axis) trials per session (x-axis) of one subject. Color code stands for the participant *Linear* adaptation time. (B) Prevalence of *CF* (y-axis) trials for subjects who practiced on set A Objects (light blue) and those who practiced on Features (pink) for each set B object at the 4th practice session (p<0.05, for all objects, Chi-square test of independence and Bootstrap). N Trials per shape: Ellipse-44 (Protocol I-27, II-17), Hexagon: 38 (Protocol I-17, II-11). L: 59 (Protocol I-34, II-25). Trapezoid: 96 (Protocol I-61, II-35), Diamond: 18 (Protocol I-10, II-8).session protocol II used significantly more *CF* in all sessions ($\chi^2_{\text{Session I}}$ (1, N=179) =17, $\chi^2_{\text{Session II}}$ (1, N=171) =36.45, $\chi^2_{\text{Session III}}$ (1, N=182) =38.95, $\chi^2_{\text{Session IV,}}$ (1, N=245) =30.94) and for all shapes(χ^2_{Ellipse} (1, N= 44) =5.23, χ^2_{Hexagon} (1, N=28) =7.33, χ^2_{L} (1, N= 59) =5.45, $\chi^2_{\text{Trapezoid}}$ (1, N=96) =13.33 χ^2_{Diamond} (1,N=18) =5.51. Error bars represent the standard error of the mean, each data point represents the prevalence of *CF* trials for one subject. (C) Visit rates of participants who practiced on Objects (top) and those who practiced on Features (bottom), during session IV. N =8 subjects, (Objects practice -5, Features practice-3).

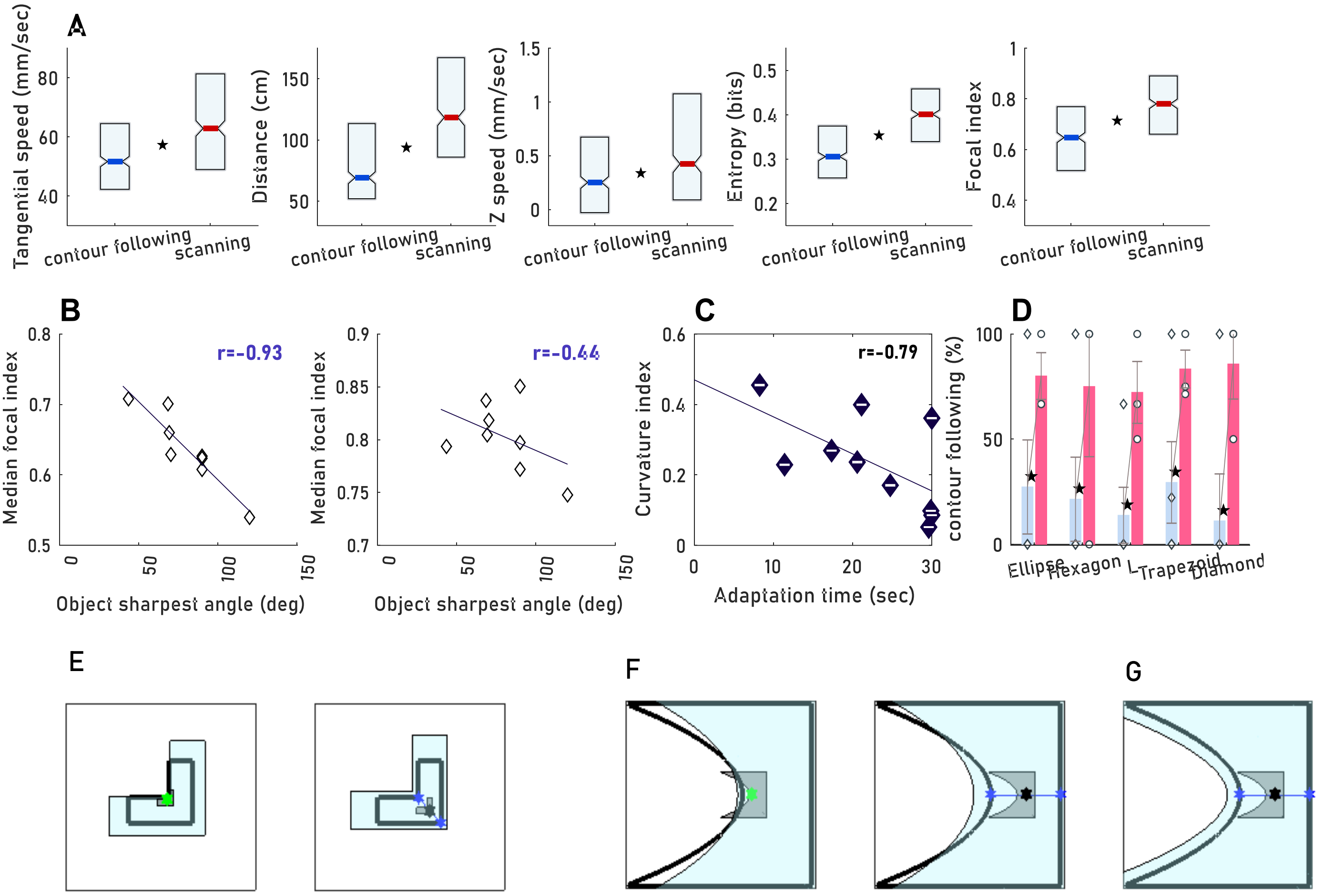

Supplementary Figure 4. (A-D), Trial classification: Results were replicated using human coders' classification: (A) *SC* and *CF* classified by the human coders differed in their mean kinematics and focal index (p<0.001, Mann–Whitney U test and Bootstrap), replicating the algorithm-based differences (Fig. 2D, Fig. 3B). (B) Objects' median focal index (y-axis) and objects' sharpest angle (x-axis) are negatively correlated for human classified *CF* (left) but not for *SC* trials (right), replicating the correlation observed using the algorithm (Fig. 3D). (C) Human-classified *CF*-trials curvature indices were highly correlated with participants' adaptation times. (r =-0.79, p =0.012, adjusted alpha=0.01, 0.05/5), as in the case of the algorithm categorization (Fig. 5F). (D) Prevalence of *CF* trials (y-axis) for subjects who practiced on set A Objects (light blue) and those who practiced on Features (pink) for each set B object at the 4th practice session. Prevalence of *CF* trials was significantly higher for those who practiced on Features (p<0.05, Chi-square test of independence and Bootstrap) as in the case of the algorithm categorization (supplementary material, fig.3). (E, F, G), Classification of L and convex trials: To allow for trial classification, a smaller area object (0.25 smaller, gray) and a larger area object (1.5 larger, light blue) were plotted around objects (black line) centroid (green). In the case of the 'L' (E, left) and 'Convex' (F, left) the centroid (green) lies close to the object contour. Therefore, different points within the object were chosen: For the 'L' a middle point (black) between two points on the contour (blue) was chosen, and the small and large objects were plotted around this point (E, right). For the 'Convex', similarly, a middle point (black) between the blue marked points was chosen (F, middle) and the small and large object were plotted around this point. For the 'Convex' feature, this definition still did not suffice for the larger object to completely contain the original shape (F, middle). Therefore, the larger object was finally obtained by replotting the object's border at a distance increased by 10 mm in the relevant direction (G).

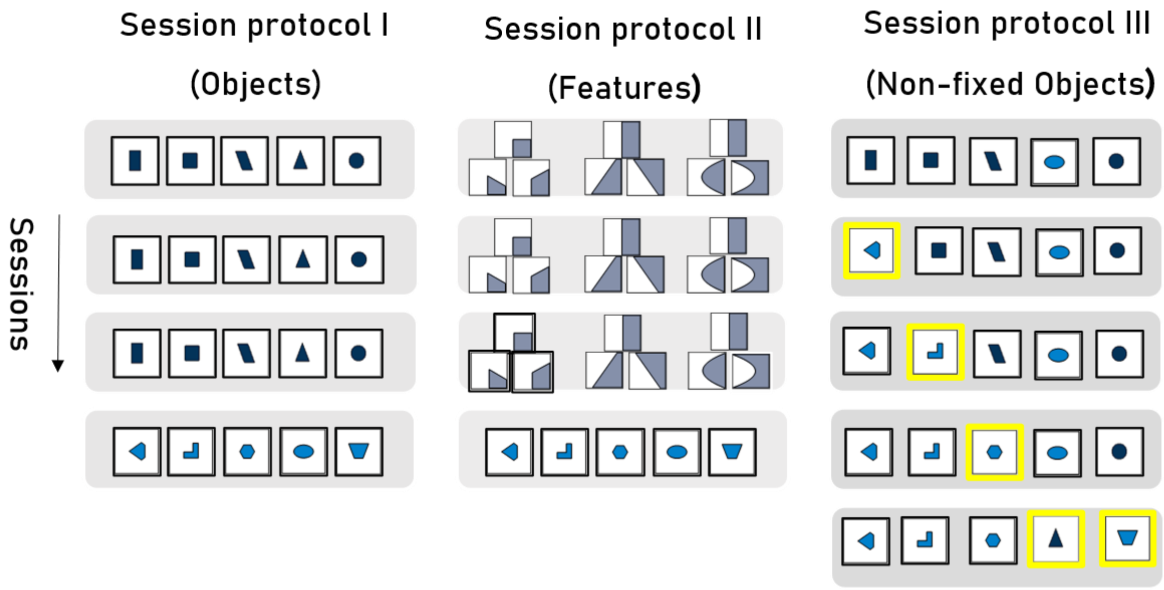

Supplementary Figure 5. Session protocols. Left, the objects presented to the participants during session protocol I. Middle, the features and objects presented to the participants during session protocol II. Right, the objects presented to the participants during session protocol III; yellow frames denote the objects that were novel in that session.
